# Sinus tract and purulence as clinical criteria for periprosthetic joint infection: diagnostic accuracy, microorganisms, and clinical outcomes

**DOI:** 10.5194/jbji-11-305-2026

**Published:** 2026-05-29

**Authors:** Markus Luger, Martin McNally, Lukas Rabitsch, Reinhard Windhager, Richard Lass, Irene Katharina Sigmund

**Affiliations:** 1 Department of Orthopaedics and Trauma Surgery, Medical University of Vienna, Spitalgasse 23, 1090 Vienna, Austria; 2 Nuffield Orthopaedic Centre, Oxford University Hospitals, Oxford, UK

## Abstract

**Introduction**: Clinical criteria are included in all current periprosthetic joint infection (PJI) definitions, with sinus tract and purulence among the most unequivocal indicators of infection. However, evidence regarding their diagnostic accuracy remains limited. This study evaluated their diagnostic performance, associated microbial profile, and clinical outcomes. **Methods**: This retrospective study included 463 revision hip and knee arthroplasties between 2015 and 2023. A total of 245 (52.9 %) were classified as infected according to the 2021 European Bone and Joint Infection Society (EBJIS) definition. Receiver-operating-characteristic (ROC) curves and their areas under the curve (AUCs) were used for diagnostic accuracy calculations. Follow-up analysis was conducted using the Kaplan–Meier survival estimator. **Results**: Sensitivities for sinus tract and purulence were 12.7 % (95 % CI: 8.8–17.5) and 35.1 % (29.1–41.4), with specificities of 100 % (98.3–100) for both. AUCs were 0.546 (0.521–0.571) and 0.695 (0.653–0.737). The highest prevalence of sinus tract was observed in *Candida albicans* (50 %), polymicrobial infection (33 %), and *Cutibacterium acnes* (30 %), whereas purulence was most prevalent in *Streptococcus* spp. (73 %), *Staphylococcus aureus* (72 %), Enterobacteriaceae (50 %), and polymicrobial infections (43 %). Both were associated with lower 3-year infection-free survival; this was significant for sinus tract ( p=0.011 ) and showed a trend for purulence ( p=0.068 ). **Conclusion**: A sinus tract can be considered a confirmatory criterion for PJI due to its excellent specificity. Although highly specific, purulence remains subjective, with limited sensitivity, and should, if considered, be regarded only as a suggestive criterion. Both parameters were associated with lower survival rates and a consistent trend towards treatment failure.

## 1 Introduction

The diagnosis of periprosthetic joint infection (PJI) is multifaceted and guided by consensus definitions from specialist societies (Parvizi et al., 2011; Osmon et al., 2013; Parvizi et al., 2014, 2018; McNally et al., 2021). These definitions incorporate a range of diagnostic modalities, including clinical findings, serum and synovial biomarkers, microbiological cultures, and histopathological evaluation. Among the clinical features, a sinus tract communicating with the prosthesis and visible purulence surrounding the prosthesis during revision surgery are most prominent. While a sinus tract is universally regarded definitive evidence of infection according to all PJI definitions (McNally et al., 2021; Parvizi et al., 2018; Osmon et al., 2013; Parvizi et al., 2011, 2014), purulence should be interpreted with greater caution. The IDSA definition considers purulence a major (confirmatory) criterion (Osmon et al., 2013). In contrast, the MSIS 2011 definition included purulence as a minor criterion (Parvizi et al., 2011), but it was excluded in the revised MSIS/ICM 2013 definition (Parvizi et al., 2014). In the ICM 2018 and EBJIS 2021 definitions, purulence was again included as a minor or suggestive criterion (McNally et al., 2021; Parvizi et al., 2018). The reason for this debate is likely that some aseptic conditions, including adverse local tissue reactions (ALTRs, frequently seen in metal-on-metal articulations, trunnionosis, or other instances with dynamic contact of metallic components) and crystal arthropathy, can present with macroscopically cloudy or turbid fluid (Trebse and Roskar, 2021; McNally et al., 2021; Alijanipour et al., 2015), making it difficult to distinguish these cases from true PJI with purulence.

However, although sinus tract and purulence are generally well recognized in clinical practice as possible indicators of PJI, evidence-based literature on their diagnostic value is still lacking. In particular, studies focusing on intraoperative purulence are scarce and often unsystematic. and lack standardized definitions. Furthermore, robust data quantifying the diagnostic accuracy of both sinus tract and purulence are currently unavailable, making it difficult for clinicians to reliably interpret these findings in routine practice. Given the clinical relevance of these signs, a detailed evaluation of their diagnostic performance is warranted.

Therefore, the aim of this study was to assess the diagnostic accuracy of the clinical signs of sinus tract and purulence surrounding the prosthesis for the confirmation of PJI in patients undergoing revision hip or knee arthroplasty. In addition, the microbial profile and patient outcomes associated with these clinical signs were determined.

## 2 Materials and methods

### 2.1 Study design

**Figure 1:**
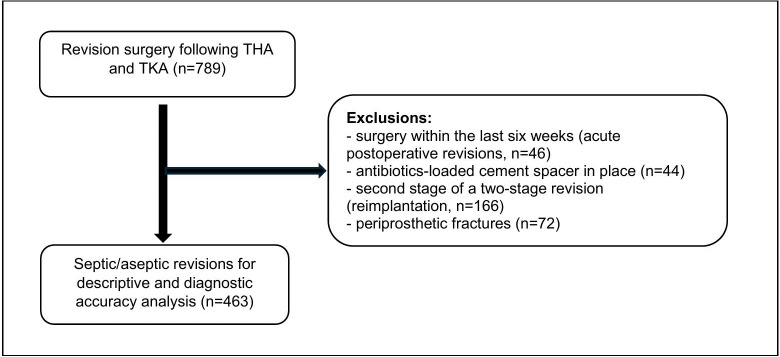
Flowchart of patient inclusion.

This retrospective cohort study was conducted at a single tertiary centre specializing in revision arthroplasty and periprosthetic joint infection. Ethical approval was obtained, and the study was performed in accordance with the principles stipulated by the Declaration of Helsinki (World Medical Association, 2013). A consecutive series of patients with revision surgery after total hip arthroplasty (THA) and total knee arthroplasty (TKA) from 2015 to 2023 was included in the analysis. Patients at the second stage of a two-stage procedure, with an antibiotic-loaded cement spacer in place, with periprosthetic fractures, and who had undergone surgery at the same joint within 4 weeks (early acute infections) were excluded. The EBJIS definition of 2021 was utilized for the diagnosis of PJI (McNally et al., 2021), and only cases classified as “infection confirmed” were included in the analysis. A standardized diagnostic workup was performed prior to and during revision surgery as specified by the definition. Demographic data; comorbidities; clinical features; and the results of serum inflammatory parameters, synovial fluid analysis, conventional cultures of synovial fluid, deep tissue samples, and sonication fluid, and histology were documented accordingly. Patients were followed up with in regular intervals after revision surgery during scheduled outpatient visits or phone calls. Figure 1 shows the recruitment and inclusion of patients. Late acute (acute haematogenous) infection was defined as symptom duration of <3 weeks following an uneventful postoperative period of >4 weeks, whereas chronic infection was defined as symptom duration of >3 weeks occurring >4 weeks after the index surgery (Sigmund et al., 2025).

### 2.2 Definitions

**Figure 2:**
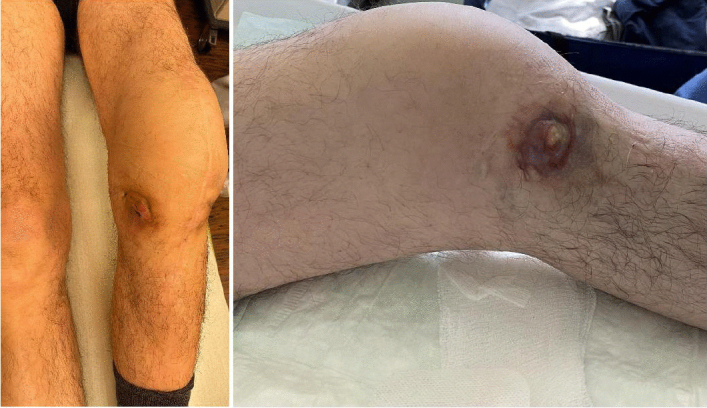
A draining sinus tract in a 63-year-old male patient with a periprosthetic joint infection of the knee caused by a multi-drug-resistant *Staphylococcus epidermidis*.

A sinus tract was defined as a fistula connecting the arthroplasty to the skin or direct visualization of the prosthesis, as reported in the outpatient, admission, or operation note (Fig. 2) (McNally et al., 2021; Sigmund et al., 2025). Prolonged postoperative wound discharge was not considered to be sinus tract (Sigmund et al., 2025). Purulence was regarded as a macroscopically turbid exudate of white to yellow or brown colour directly surrounding the arthroplasty in the periprosthetic tissue (Alijanipour et al., 2015; Trebse and Roskar, 2021; McNally et al., 2021) and documented by the surgeon in the operative report. Cloudy fluid associated with ALTR and/or crystal arthropathy cases was not considered to be purulence (McNally et al., 2021; Trebse and Roskar, 2021). Culture positivity was determined based on both preoperative and intraoperative microbiological findings. Preoperative antibiotics were defined as any systemic antibiotic therapy administered within 14 d prior to surgery. Treatment success was defined according to the Delphi international multidisciplinary consensus criteria (Diaz-Ledezma et al., 2013), with the additional requirement that no suppressive antibiotic therapy was necessary.

### 2.3 Statistical analysis

Statistical analysis was performed using RStudio version 2025.05.1 (Posit PBC, Boston, USA [R version 4.5.0]). Categorial variables are given as absolute and relative frequencies, and continuous variables are given as median and interquartile ranges (IQRs). The Chi-squared test, Student's t test, and Fisher's exact test were used wherever appropriate. For diagnostic accuracies, receiver-operating-characteristic (ROC) curves were utilized, and their areas under the curve (AUCs) were calculated using the EBJIS definition as the gold-standard reference. To avoid incorporation bias, sinus tract was excluded from the infection definition. Purulence is not considered to be a confirmatory criterion in the EBJIS definition. The infection-free survival rates were assessed using the Kaplan–Meier estimator. Statistical significance was set at p<0.05 .

## 3 Results

### 3.1 Demographics

In total, 463 patients were included (Fig. 1), of whom 245 (52.9 %) were identified as septic using the EBJIS definition (Table 1). A total of 265 (57.2 %) were female, and the median age was 74 years (IQR: 64.0–81.0). A total of 214 cases (46.2 %) were hips, and 249 were knees (53.8 %) (Table 2). In the septic cohort, significantly more patients had an American Society of Anaesthesiologists (ASA) Score of 3 or more (62.0 % vs. 47.2 %, p=0.002 ). A total of 44 of 245 septic cases (18 %) received preoperative antibiotics ( p<0.001 ). Metallosis and other types of wear were encountered significantly more often in aseptic cases ( p=0.002 and p=0.001 ). Late acute infections showed a significantly higher incidence of purulence compared to chronic infections ( p<0.001 ).

**Table 1:** Demographics of included patients. Bold font indicates statistical significance ( p<0.05 ).

Demographics	Total ( n=463 )	Septic cases ( n=245 )	Aseptic cases ( n=218 )	p -value
Median age, years (IQR)	74.0 (64.0–81.0)	75.0 (65.0–81.0)	74.0 (63.0–80.0)	0.819^a^
Female sex, n (%)	265 (57.2)	129 (52.7)	136 (62.4)	**0.035** ^b^
Median BMI (IQR)	27.8 (24.1–32.4)	28.3 (24.1–33.0)	27.6 (24.0–32.0)	0.236^a^
ASA grade ≥ 3, n (%)	253 (55.0)	150 (62.0)	103 (47.2)	**0.002** ^b^
Localization				
Hip, n (%)	214 (46.2)	113 (46.1)	101 (46.3)	0.964^b^
Knee, n (%)	249 (53.8)	132 (53.9)	117 (53.7)	
Preoperative antibiotics, n (%)	46 (9.9)	44 (18.0)	2 (0.9)	$<0.001^{c}$
Rheumatoid arthritis, n (%)	28 (6.0)	14 (5.7)	14 (6.4)	0.750^b^
ALTR, n (%)				
Metallosis, n (%)	36 (7.8)	10 (4.1)	26 (11.9)	**0.002** ^b^
Other wear, n (%)	13 (2.8)	1 (0.4)	12 (5.5)	**0.001** ^c^
Sinus tract, n (%)	31/463 (6.7)	31/245 (12.7)	0/218 (0.0)	$<0.001^{c}$
Intraoperative purulence, n (%)	86/463 (18.6)	86/245 (35.1)	0/218 (0.0)	$<0.001^{c}$
Median CRP, mg L^−1^ (IQR)	8.5 (2.4–50.2)	37.1 (9.5–139.1)	2.9 (1.2–7.1)	$<0.001^{a}$
≥ one culture(s) positive, n (%)	187 (40.5)	168 (68.6)	23 (10.6)	$<0.001^{b}$

^a^ Independent-sample t test, ^b^ Chi-squared test, ^c^ Fisher's exact test. IQR: interquartile range; BMI: body mass index; ASA: American Society of Anaesthesiologists; ALTR: adverse local tissue reaction; CRP: C-reactive protein; SF: synovial fluid; WBC: white blood cell count; %PMN: percentage of polymorphonuclear neutrophils

**Table 2:** Distribution of localization (hip, knee) and timing (acute, chronic) of periprosthetic joint infection with sinus tract and intraoperative purulence. Bold font indicates statistical significance ( p<0.05 ).

Parameter	Hip	Knee	p value	Late acute infections	Chronic infections	p value	Total
	( n=114 )	( n=131 )		( n=80 )	( n=165 )		( n=245 )
Sinus tract, n (%)	19/114 (16.7)	12/131 (9.2)	0.116^*^	0/80 (0.0)	31/165 (18.8)	$<0.001^{*}$	31/245 (12.7)
Purulence, n (%)	35/114 (30.7)	51/131 (38.9)	0.226^*^	42/80 (52.5)	44/165 (26.7)	$<0.001^{*}$	86/245 (35.1)

^*^ Chi-squared test.

### 3.2 Diagnostic accuracies

**Figure 3:**
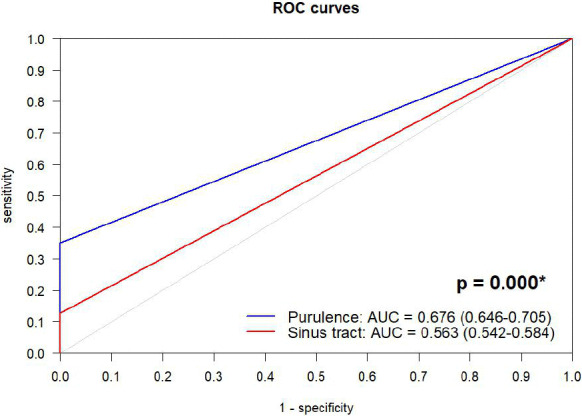
Receiver-operating-characteristic (ROC) curves for accuracy of sinus tract and purulence in diagnosis of periprosthetic joint infection. AUC: area under the curve. ^*^ DeLong test.

**Figure 4:**
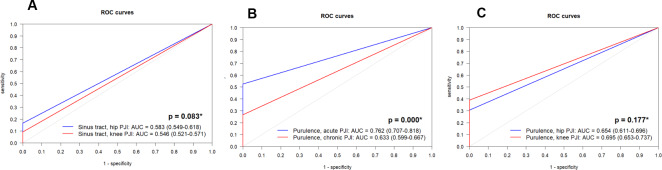
Receiver-operating-characteristic (ROC) curves for accuracy of **(A)** sinus tract in hip and knee PJI, **(B)** intraoperative purulence in acute and chronic PJI, and **(C)** intraoperative purulence in hip and knee PJI. AUC: area under the curve. ^*^ DeLong test.

Sensitivities of sinus tract and purulence were 12.7 % (8.8–17.5) and 35.1 % (29.1–41.4), while both demonstrated ideal specificities of 100 % (98.3–100), with no false-positive case. AUC of purulence was significantly higher than that of sinus tract (0.676 (0.646–0.705) vs. 0.563 (0.542–0.584), p<0.001 , Fig. 3). Furthermore, purulence demonstrated better accuracy in acute PJI (0.762 (0.707–0.818)) compared with chronic PJI (0.633 (0.599–0.667); p<0.001 ). No significant differences were observed in the localization of sinus tract or purulence between hips and knees ( p>0.05 , Fig. 4). Diagnostic performance metrics, including subgroup analysis, are given in Table 3.

**Table 3:** Diagnostic accuracies of sinus tract and purulence for diagnosing periprosthetic joint infections.

Parameter	Sensitivity	Specificity	Youden's	PPV	NPV	LR +	LR -	AUC
	(%)	(%)	index	(%)	(%)			
Sinus tract	12.7 (8.8–17.5)	100 (98.3–100)	0.127	100 (88.8–100)	50.5 (45.6–55.3)	– (–)	0.874 (0.723–1.055)	0.563 (0.542–0.584)
Sinus tract (hip)	16.7 (10.3–24.8)	100 (98.3–100)	0.167	100 (82.4–100)	69.7 (64.2–74.7)	– (–)	0.833 (0.655–1.060)	0.583 (0.549–0.618)
Sinus tract (knee)	9.2 (4.8–15.5)	100 (98.3–100)	0.092	100 (73.5–100)	64.7 (59.3–69.8)	– (–)	0.908 (0.727–1.136)	0.546 (0.521–0.571)
Purulence	35.1 (29.1–41.4)	100 (98.3–100)	0.351	100 (95.8–100)	57.8 (52.7–62.9)	– (–)	0.649 (0.529–0.796)	0.676 (0.646–0.705)
Purulence (acute infections)	52.5 (41.0–63.8)	100 (98.3–100)	0.525	100 (91.6–100)	85.2 (80.2–89.3)	– (–)	0.475 (0.337–0.670)	0.762 (0.707–0.818)
Purulence (chronic infections)	26.7 (20.1–34.1)	100 (98.3–100)	0.267	100 (92.0–100)	64.3 (59.0–69.4)	– (–)	0.733 (0.587–0.916)	0.633 (0.599–0.667)
Purulence (hip)	30.7 (22.4–40.0)	100 (98.3–100)	0.307	100 (90.0–100)	73.4 (68.0–78.3)	– (–)	0.693 (0.536–0.896)	0.654 (0.611–0.696)
Purulence (knee)	38.9 (30.5–47.8)	100 (98.3–100)	0.389	100 (93.0–100)	73.2 (67.7–78.1)	– (–)	0.611 (0.473–0.789)	0.695 (0.653–0.737)

PPV: positive predictive value; NPV: negative predictive value; LR + : positive likelihood ratio; LR - : negative likelihood ratio; AUC: area under the curve.

### 3.3 Microorganisms

Of the 245 septic cases, 168 yielded positive cultures (69.0 %, Table 4). *Staphylococcus aureus* was the most frequently isolated pathogen, detected in 47 patients (19.2 %). Coagulase-negative *staphylococcus* was found in 37 patients (15.1 %), *Streptococcus* spp. was found in 15 (5.7 %), and *Cutibacterium* spp. was found in 10 patients (4.1 %). A total of 30 PJI cases (12.2 %) were polymicrobial infections.

**Table 4:** Microbial profile of septic cases, including infections with sinus tract and purulence.

Microorganisms	Septic cases	Sinus tract	Purulence
	( n=245 )	( n=31 )	( n=86 )
*Staphylococcus aureus*, n (%)	47 (19.2)	6/47 (12.8)	34/47 (72.3)
Coagulase-negative staphylococci, n (%)	37 (15.1)	2/37 (5.4)	2/37 (5.4)
*Streptococcus* spp., n (%)	15 (5.7)	0/14 (0.0)	11/15 (73.3)
*Cutibacterium* spp., n (%)	10 (4.1)	3/10 (30.0)	0/10 (0.0)
Enterobacteriaceae, n (%)	8 (3.3)	1/8 (12.5)	4/8 (50.0)
*Enterococcus* spp., n (%)	7 (2.9)	0/7 (0.0)	0/7 (0.0)
*Candida albicans*, n (%)	4 (1.6)	2/4 (50.0)	1/4 (25.0)
*Pseudomonas aeruginosa*, n (%)	2 (0.8)	0/2 (0.0)	0/2 (0.0)
Other^*^, n (%)	9 (3.7)	1/9 (11.1)	2/9 (22.2)
Polymicrobial, n (%)	30 (12.2)	10/30 (33.3)	13/30 (43.3)
Culture-negative, n (%)	76 (31.0)	6/76 (7.9)	19/76 (25.0)

Other^*^: *Finegoldia magna* ( n=2 ), *Corynebacterium* spp., *Actinomyces neuii*, *Bacteroides thetaiotaomicron*, *Micrococcus luteus*, *Mycobacterium* spp., *Parvimonas micra*, *Pasteurella multocida*.

Among pathogens with ≥ 10 cases, polymicrobial infections ( n=10/30 , 33.3 %), *Cutibacterium* spp. ( n=3/10 , 30.0 %), and *Staphylococcus aureus* ( n=6/47 , 12.8 %) most frequently led to sinus tract formation. Among less common pathogens ( <10 cases), *Candida albicans* was the most prevalent pathogen ( n=2/4 , 50 %) causing a sinus tract.

A total of 86 PJI cases (35.1 %) presented with purulence surrounding the prosthesis. Pathogens with the highest prevalence were *Streptococcus* spp. ( n=11/15 , 73.3 %), *Staphylococcus aureus* ( n=34/47 , 72.3 %), and polymicrobial infections ( n=13/30 , 43.3 %; Table 4). The culture-negative rate in sinus tract group was 19 % ( n=6/31 ), and, in the purulence group, it was 22 % ( n=19/86 ).

### 3.4 Follow-up analysis

**Figure 5:**
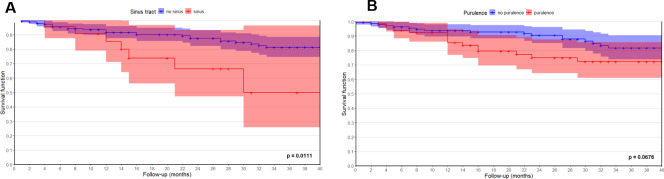
Kaplan–Meier curves showing infection-free survival up to 40 months after reimplantation in cases with and without **(A)** a sinus tract and **(B)** purulence. Note that ^+^ indicates censored data. ^*^ Log-rank test.

In the entire study cohort, infection-free survival rates were 90.9 % (95 % CI: 86.8–95.1) at 1 year, 85.2 % (79.7–90.7) at 2 years, and 78.5 % (71.5–85.4) at 3 years. Patients with concomitant sinus tract had significantly lower infection-free survival compared to those without ( p=0.011 , log-rank test). In sinus tract group, infection-free survival was 85.3 % (71.1–100) at 1 year and 49.8 % (25.7–96.4) at 3 years versus 91.6 % (87.5–95.9) and 81.3 % (74.6–88.5) in the group without sinus tract. A similar trend was observed for cases with intraoperative purulence, although this did not reach statistical significance ( p=0.068 ). In these patients, infection-free survival was 85.3 % (76.9–94.7) at 1 year and 72.3 % (60.9–85.7) at 3 years compared to 93.8 % (89.7–98.1) and 81.7 % (73.8–90.5) in patients without purulence (Table 5, Fig. 5).

**Table 5:** Survival rates of infected cases with and without a sinus tract and purulence. Bold font indicates statistical significance ( p<0.05 ).

Survival distribution	Sinus tract	No sinus tract	p value	Purulence	No purulence	p value
function	( n=23 )	( n=192 )		( n=71 )	( n=144 )	
6 months, % (CI)	95.7 (87.7–100)	95.5 (92.5–98.6)	**0.011** ^*^	94.0 (88.4–99.9)	96.3 (93.1–99.5)	0.068^*^
12 months, % (CI)	85.3 (71.1–100)	91.6 (87.5–95.9)		85.3 (76.9–94.7)	93.8 (89.7–98.1)	
18 months, % (CI)	73.8 (56.3–96.8)	90.1 (85.6–94.9)		79.5 (69.7–90.7)	92.8 (88.3–97.5)	
24 months, % (CI)	66.4 (47.2–93.4)	87.5 (82.3–93.0)		74.9 (64.2–87.5)	90.5 (85.1–96.1)	
30 months, % (CI)	49.8 (25.7–96.4)	84.6 (78.8–91.0)		72.3 (60.9–85.7)	86.4 (79.8–93.6)	
36 months, % (CI)	49.8 (25.7–96.4)	81.3 (74.6–88.5)		72.3 (60.9–85.7)	81.7 (73.8–90.5)	

^*^ Log-rank test. CI: 95 % confidence-interval.

## 4 Discussion

In our study, the presence of a sinus tract communicating with the prosthesis or joint demonstrated an ideal specificity of 100 % (95 % CI: (98.3–100)) but a low sensitivity of 12.7 % (8.8–17.5; sinus tract). Although included in all infection definitions as a major or confirmatory criterion for PJI (McNally et al., 2021; Osmon et al., 2013; Parvizi et al., 2011, 2014, 2018), to our knowledge, no evidence to date has reported on the diagnostic value of a sinus tract. Based on our results, this clinical sign confirms infection, but its absence does not exclude PJI, as has been previously recognized but never accurately analysed. Accordingly, the presence of a sinus tract can therefore continue to be recommended as a confirmatory criterion for diagnosing PJI.

However, the definition of a sinus tract remains an important yet debated aspect in PJI diagnosis. Some consider prolonged postoperative wound drainage as a sinus tract, whereas others restrict the term to a chronic infection characterized by an epithelium-lined channel connecting the prosthesis to the skin. A clear distinction between transient wound leakage and a true sinus tract with a fully developed epithelialized wall is essential. Infection-induced inflammation can lead to accumulation of synovial fluid within the joint. When intra-articular pressure increases, this fluid may follow the path of least resistance to the skin, forming an immature tract. Persistent infection with ongoing fluid production can promote epithelialization, resulting in a mature sinus tract capable of continuous drainage. A study on chronic sinuses as therapy for treatment-resistant PJIs has shown that creating an iatrogenic tract with a large-diameter drainage tube requires several weeks (6 weeks) for stabilization and epithelialization (Klim et al., 2023), emphasizing that a mature sinus tract develops only over a prolonged time. In chronic infections, the slow and persistent accumulation of fluid under low pressure allows epithelialization and biofilm maturation, marking a true sign of chronic infection. In the present cohort, sinus tracts were observed exclusively in chronic infections. In contrast, skin breakdown or short-term wound leakage in the early postoperative period typically represents immature or incomplete tracts (resulting from the surgical procedure and wound closure) that may reopen under minimal pressure and do not indicate a fully established sinus tract or mature biofilm. In addition, non-infectious factors, such as anticoagulation therapy or malnutrition, can also contribute to postoperative wound drainage (Chotanaphuti et al., 2019). Therefore, a true sinus tract should be strictly defined as a chronic, epithelium-lined channel connecting the prosthesis or joint to the skin, as applied in this study. Nevertheless, we acknowledge that the clinical distinction between prolonged postoperative wound drainage and early sinus tract formation remains challenging and is still subject to ongoing debate. Therefore, patients with early postoperative infections were excluded from the present study in order to minimize potential bias related to postoperative wound drainage.

Importantly, sinus tract formation can occur with any pathogen, depending on microbial virulence, the host immune response, and the surrounding soft tissue envelope. However, certain pathogens were more frequently associated with sinus tract development. In this cohort, the most common pathogens leading to sinus tract formation were *Candida albicans* (50 %), polymicrobial infections (32 %), *Cutibacterium* spp. (30.0 %), and *Staphylococcus aureus* (13 %). Higher prevalences have been reported in previous studies by Luo et al. (2025) and Budin et al. (2024), who analysed the microbial distribution in knee and hip PJI. In knee PJI, polymicrobial infections (42 %), *Staphylococcus aureus* (24 %), and *Enterococcus faecalis* (13 %) were most common (Luo et al., 2025), whereas, in hip PJI, polymicrobial (48 %), *Staphylococcus aureus* (31 %), and *Staphylococcus epidermidis* (27 %) predominated (Budin et al., 2024). Moreover, polymicrobial infections and Gram-negative bacteria have been previously linked to sinus tract formation (Tan et al., 2016; Xu et al., 2019). In our cohort, however, the number of *Enterococcus* spp. and Gram-negative infections was small ( n=7 ), precluding meaningful comparisons for these pathogens.

In previous studies, the presence of a sinus has been a predictor of treatment failure (Kheir et al., 2018; Xu et al., 2024, 2019), which is in line with our findings. Patients with a sinus tract formation showed significantly lower survival rates (49.8 % vs. 81.3 % at 36 months, p=0.011 ) compared with patients without. This striking difference underscores the challenges of managing infections with compromised soft tissue, regardless of whether a one-stage or two-stage revision is performed (Thakrar et al., 2019; Xu et al., 2019). Adequate soft tissue coverage, often with a muscle flap, is frequently required to achieve infection eradication. In our cohort, four patients ( n=4/31 , 13 %) with knee PJI underwent reconstruction using a local medial gastrocnemius flap. Three proceeded to reimplantation; one later required amputation, while the remaining two remained free of reinfection at 2-year follow-up. These cases illustrate the complexity and difficulty of managing PJI with poor soft tissue conditions, particularly around the knee.

Regarding purulence surrounding the prosthesis, an excellent specificity of 100 %, comparable to that of sinus tract, was observed in our study. Sensitivity remained higher but still insufficient at 35 %. Overall, evidence on the diagnostic accuracy of intraoperative purulence remains limited. Shohat et. al. identified purulence as an important intraoperative marker for chronic PJI in their logistic regression model (Shohat et al., 2019). In contrast, Alijanipour et. al. reported a sensitivity of 82 % but a specificity of only 32 % based on the ICM definition of 2013 (Parvizi et al., 2014). Although the authors acknowledged the limitations of purulence, they did not exclude confounding conditions (such as ALTR, inflammatory arthopathy). Furthermore, their definition of purulence differed from ours as pus was assumed to be present when the surgical findings appeared to be compatible with infection in their study (Alijanipour et al., 2015). By excluding confounding conditions and relying solely on the surgeon's explicit documentation of purulence in the operative report, we observed no false-positive cases in our cohort, aligning with a review on the interpretation of PJI diagnostic tests (Trebse and Roskar, 2021). The authors concluded that, once confounders are excluded, purulence is no longer a controversial criterion and approaches a specificity of 100 %. This underlines its value as an indicator of PJI, particularly in acute infections caused by highly virulent microorganisms. However, we acknowledge that distinguishing true purulence from merely turbid synovial fluid can be challenging. While frank pus is generally unmistakable, cloudy or opaque fluid may also occur in several aseptic conditions, including ALTR, trunnionosis, and crystal or inflammatory arthropathy (McNally et al., 2021; Tande and Patel, 2014; Trebse and Roskar, 2021; Archibeck et al., 2001). Some reports describe ALTR-associated joint effusions as thick, cloudy, “milk-stained”, or yellow-green in appearance (Mikhael et al., 2009; Browne et al., 2010), and similar findings have been observed with other bearing materials (Bonnaig et al., 2011). Kim et al. (2012) also reported yellowish opaque effusion in osteonecrosis of the femoral head. Thus, the presence of turbid fluid around prosthetic components is a characteristic but not a pathognomonic sign of infection. Therefore, in situations where uncertainty exists (such as ALTR or crystal or inflammatory arthropathies), it may, if considered at all, be more appropriately regarded as a suggestive rather than a confirmatory criterion.

In our cohort, the pathogens most frequently associated with purulence were *Streptococcus* spp. (73 %), *Staphylococcus aureus* (72 %), *Enterobacteriaceae* (50 %), and polymicrobial infections (43 %). Only Alijanipour et al. (2015) have previously examined microbial patterns in PJI cases presenting with purulence. They reported *Streptococcus* spp. (88 %) and *Staphylococcus aureus* (85 %) as the predominant pathogens associated with pus, which is consistent with our findings.

To our knowledge, this is the first study to report infection-free survival in relation to the presence of purulence. This clinical sign demonstrated a trend towards lower survival rates, although statistical significance was not reached (3-year follow up: 72 % with purulence vs. 82 % without, p=0.068 ). These findings highlight the need for meticulous deep tissue sampling, surgical debridement, and irrigation in cases presenting with purulence in order to maximize the likelihood of infection eradication and to improve outcomes.

This study is not without limitations. Due to its retrospective character, some variables were missing in certain cases. In particular, the presence of a sinus tract or purulence may be underreported in outpatient, admission, and operation notes. Standardized documentation of intraoperative findings such as sinus tract formation and purulence may improve consistency, reproducibility, and comparability across studies and institutions. The absence of a universally accepted definition of purulence introduces additional variability as its assessment depends on the surgeon's subjective interpretation. Moreover, in cases with a clear preoperative diagnosis of infection, detection bias may influence intraoperative documentation. However, these limitations reflect the everyday challenges of interpreting sinus tracts and purulence and are therefore important to evaluate in this study.

To our knowledge, this is the first study to analyse diagnostic accuracies, microbial profiles, and outcome of clinical criteria in detail, namely sinus tract and purulence surrounding the prosthesis. While there is some literature on sinus tract formation, sufficient evidence on purulence in PJI is almost completely lacking. Larger, prospective studies are necessary to further validate and refine our findings.

## 5 Conclusion

While the diagnostic value of a sinus tract has long been recognized and applied in clinical practice for PJI, robust evidence has been lacking. Our study confirms that a sinus tract communicating with the prosthesis exhibits high specificity and can therefore be recommended as a confirmatory criterion for PJI. Similarly, purulence demonstrated excellent specificity, although distinguishing true purulence from turbid synovial fluid can be challenging in clinical practice. Therefore, purulence may, if considered at all, be most appropriately regarded to be a suggestive criterion for PJI.

Sinus tracts were frequently associated with polymicrobial and fungal infections, whereas purulence was most commonly observed in infections caused by high-virulent microorganisms, such as *Streptococcus* spp. and *Staphylococcus aureus*. Both sinus tract and purulence were associated with less favourable outcomes, underlining their clinical relevance in the management of PJI.

## Data Availability

All data generated or analysed in this original article are included in the published article (see Tables 1–5 and Figs. 1–5).
